# The in vitro effect of guaraná (Paullinia cupana) extract on human peripheral blood mononuclear cells exposed to a high glucose level

**DOI:** 10.1186/1758-5996-7-S1-A227

**Published:** 2015-11-11

**Authors:** Alessandro Meneghetti Anversa, Felipe Rogalski, Fernanda Barbisan, Charles E Assman, Verônica Farina Azzolin, Andressa Duarte Seehaber, Dianni Capeletto, Gustavo Cardenas, Beatriz da Silva Rosa, Ivana Beatrice Mânica da Cruz

**Affiliations:** 1 Universidade Federal de Santa Maria, Santa Maria, Brazil

## Background

Nowadays, Type 2 Diabetes Mellitus (T2D) affects a significant percentage of the world population. Due to the high levels of glucose, it causes several damages to the cells and, consequently, to the organism. Plants, such as guaraná (Paullinia cupana), which present bioactive compounds, could help somehow in the treatment of diabetes and other chronic diseases. Studies have shown that guaraná extract, rich in compounds like methylxanthines, theobromine and theophylline, presents many beneficial effects to the organism, such as antioxidant capacity, modulation of nitric oxide intracellular levels, as well as anti-obesity and anti-inflammatory capacity.

## Objective

The aim of this study was to evaluate the in vitro effect of guaraná (Paullinia cupana) extract on the viability and cellular proliferation of Peripheral Blood Mononuclear Cells (PBMC) exposed to a high glucose level.

## Materials and methods

Guaraná powder was provided by EMBRAPA, located in the city of Maués, Amazonas. The hydroalcoholic extract was prepared following Bittencourt et al. (2013). Initially, peripheral blood from 20 young and healthy individuals (8 h of fasting) was collected. The PBMC were isolated with Ficoll-Histopaque®, cultured in RPMI 1640 medium for 72 h at 37^o^ C and treated with the guaraná concentrations (1 mg/mL, 5 mg/mL and 10 mg/mL), glucose (15 mg/mL) or both. The control group does not received any treatment. MTT assay was performed to evaluate the cellular proliferation and PicoGreen assay for the evaluation of the cellular viability.

## Results

**Figure 1:**
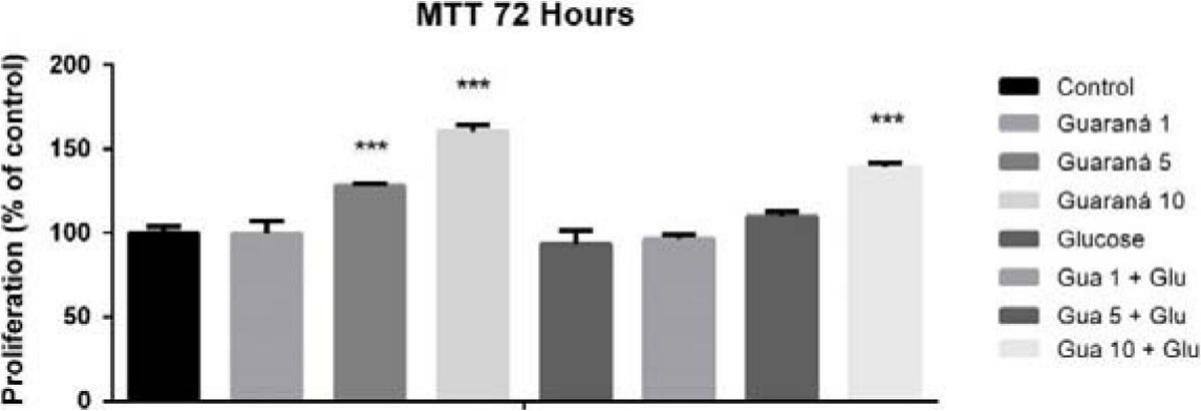
Effect of the treatment of guaraná extract and glucose at different concentrations (mg/mL) on the cellular proliferation of PBMC.

**Figure 2:**
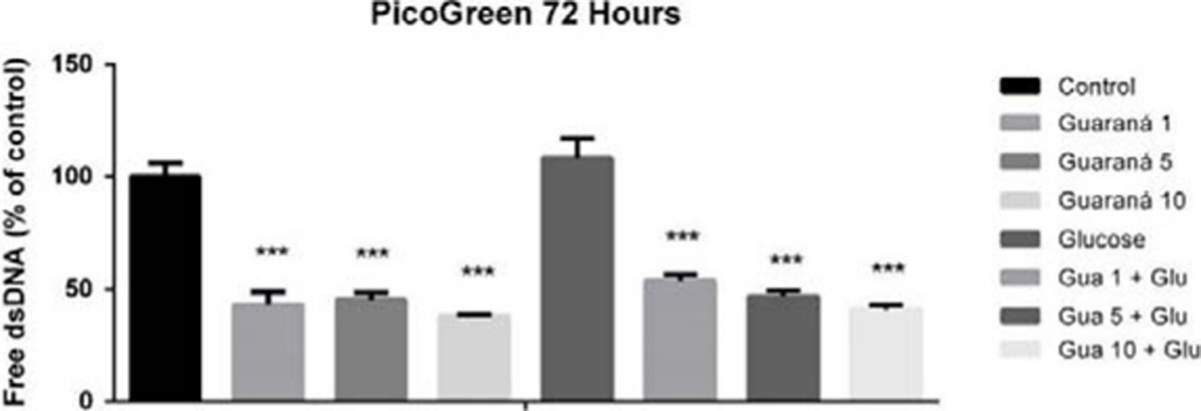
Effect of the treatment of guaraná extract and glucose at different concentrations (mg/mL) on the cellular viability of PBMC.

Guaraná increased the cellular proliferation in 50% at the 10mg/mL concentration, for both cells, treated and untreated with glucose (Fig. 1). Guaraná presented a cytoprotective effect on the cellular viability at all tested concentrations, including the cells treated with glucose (Fig. 2).

## Conclusion

The results indicate that the guaraná (P. cupana) extract has a beneficial effect on the PBMC, increasing the cellular proliferation and decreasing the extracellular dsDNA levels. These data corroborate with other studies that have shown the guaraná antioxidant capacity. The guaraná extract presented statistically significant positive activity even when combined with a high level of glucose, indicating that it could be used to improve the organism functioning of healthy people as well as patients with T2D.

